# DNA Damage Response Signals Transduce Stress From Rheumatoid Arthritis Risk Factors Into T Cell Dysfunction

**DOI:** 10.3389/fimmu.2018.03055

**Published:** 2018-12-20

**Authors:** Lan Shao

**Affiliations:** The Center for Translational Medicine, The First Affiliated Hospital, Sun Yat-sen University, Guangzhou, China

**Keywords:** DNA damage response, ataxia telangiectasia mutated, rheumatoid arthritis, high-risk factors, T cell dysfunction

## Abstract

Rheumatoid arthritis (RA) is an autoimmune-mediated disease that is associated with significant cartilage damage and immunosenescence. Despite decades of research, the major signal pathways that initiate RA are still unclear. The DNA damage response (DDR) is a specific and hierarchical network that includes cell cycle checkpoints, DNA repair, and DNA-damage tolerance pathways. Recent studies suggest that this condition is associated with deficits in telomere maintenance and overall genomic instability in the T cells of RA patients. Analysis of the underlying mechanisms has revealed defects in DDR pathways. Particularly, the DNA repair enzyme, ataxia telangiectasia mutated (ATM), is downregulated, which leaves the damaged DNA breaks in RA-associated T cells unrepaired and pushes them to apoptosis, exhausts the T cell pool, and promotes the arthritogenesis effector function of T cells. This review discusses recent advancements and illustrates that risk factors for RA, such as viral infections, environmental events, and genetic risk loci are combat with DDR signals, and the impaired DDR response of RA-associated T cells, in turn, triggers disease-related phenotypes. Therefore, DDR is the dominant signal that converts genetic and environmental stress to RA-related immune dysfunction. Understanding the orchestration of RA pathogenesis by DDR signals would further our current knowledge of RA and provide novel avenues in RA therapy.

## Introduction

The human genome is subjected to constant endogenous and environmental assaults. To maintain genomic integrity, cells have evolved a highly complex signal transduction network to recognize, signal, and repair DNA lesions. A chief component of DNA damage is PI3-kinase related protein kinases (PIKKs), including ataxia telangiectasia mutated (ATM), DNA-PKcs, and ATR(1). These enzymes orchestrate hierarchical pathways that protect genomic integrity, which is termed the DNA damage response (DDR). RPA-coated single-stranded DNA (ssDNA) activates ATR and double-stranded breaks (DSBs), which are particularly hazardous to the cells repaired by ATM and DNA-PK.When DSBs occur, the breaks are recognized by the MRN complex (MRE11A-Rad50-NBS1) and then ATM is recruited to the damage site (1, 2). Activated ATM orchestrates the DNA repair process by phosphorylating multiple proteins, such as H2AX and 53BP1, to form characteristic DNA foci (3). ATM also phosphorylates the substrates CHK2 and p53 to initiate cell-cycle arrest (4). This method of DNA repair is called the homologous recombination (HR) repair pathway. Alternatively, DSBs activate non-homologous end joining (NHEJ) repair pathway which mediated by DNA-PKcs (5). Upon detecting DNA damage, the cell follows two routes: activation of DDR-dominant cell cycle checkpoints and cell cycle arrest to facilitate repair, or if damaged beyond repair, induction of apoptosis.

Persistent DNA damage signals are considered a major contributor to cellular senescence and organismal aging (6). Immune aging is a central component of advancing age and leads to the loss of immune system-mediated protection against infections, and is a prominent risk factor for chronic inflammation and the pathogenesis of autoimmune diseases (7–9). Rheumatoid arthritis (RA) is an autoimmune disorder characterized by accelerated immune senescence (10–15). T cells from RA patients appear prematurely-aged and exhibit accumulation of CD28^−^ effector T cells, shortened telomeres, DNA damage accumulation, as well as an excess production of proinflammatory cytokines, all of which are compatible with normal cellular senescence. Interestingly, however, T cells in RA patients lack an irreversible cell cycle arrest and are deficient in the characteristic DNA repair foci; these are considered cardinal features of cellular senescence. In contrast, RA-associated T cells have a suppressed DDR pathway and a disrupted G2/M cell cycle checkpoint (10). Consequently, cells are hyperproliferative, more vulnerable to apoptosis, and clonal expansion is decreased in naïve RA T cells (14). All those studies indicate that the DDR pathway is likely associated with RA pathogenesis-related T cell dysfunction.

In this review, we discuss the complex association between the DDR pathway and T cell pathogenesis in RA. Particularly, we focus on the mechanisms underlying the effects of RA risk factors on DDR pathway and the role of modified DDR signals in RA-mediated T cell dysfunction. Determination of the molecular role of the DDR pathway in RA-associated T cell evolution may facilitate our understanding and management of this disease.

## RA Risk Factors Lead to Defects in DDR Signaling

T cells have a long life in circulation and are exposed to various environmental, pathogenic, and genetic stresses, which decrease their survival time. Consequently, cells require a common pathway to collectively combat endogenous and extracellular stresses. This usually occurs before the onset of RA, thus indicating a preclinical phase of the disease. Multiple high-risk factors for RA are also triggers for DDR signals (16, 17). This section discusses how cellular DDR pathways serve as the major sensors in T cells to combat endogenous and environmental stresses, and how they may be modified from their original course.

### Genetic Factors

Multiple genetic foci have been identified as risk factors of RA. *HLA-DRB10401* and *HLA-DRB 0404* are the alleles most strongly associated with RA (18–20). Analyses of telomeric lengths in CD4^+^ T cells from RA patients showed that HLA-DR-B1^*^04 is sufficient to accelerate telomere shortening (21), suggesting that HLA-DR-B1^*^04 affects signals regulating telomere maintenance. Additionally, genome-wide association studies (GWASs) have identified more than 100 common single nucleotide polymorphisms (SNPs) for RA risk, including *ATM, PTPN22, CTLA-4, TRAFs, PADI4*, and *STAT4* (22–26).

How are these genes involved in the regulation of DDR signals? ATM is the important element for DDR signals; *ATM* polymorphism contribute to RA development by affecting the efficiency of DDR repair. Moreover, ATM function is directly regulated by PTPN22 (27). The interaction between ^*^T1858 allele of *PTPN22* and polymorphism of ^*^Pro allele of the *p53* codon 72 strongly increase the autoimmune inflammatory (28). The key downstream target of CTLA-4 is Akt, which is also an upstream signal for ATM (29). Members of the TRAFs family are involved in DNA damage-induced NF-κB activation. After DNA damage, ATM is translocated to the cytosol and interacts with TRAF6 to form ATM-TRAF6-cIAP1 complex, which catalyzes the monoubiquitination of NEMO to activate genotoxic NF-κB activation (30). The PADI4, a citrullination enzyme, is critical for anti-citrullinated peptide antibodies (ACPA) production in RA. PADI4 has been reported to citrullinatic modification of multiply proteins in a p53/PADI4-dependent manner (31, 32). STAT4 is a strong responder to DDR signals. The *STAT4* SNPs exert synergistic effects with DDR signals to mediate citrullination production in the T cells of RA patients (33). Further analysis suggests that more genetic risk factors for RA could be included in the network of DDR signals, functioning either upstream of DDR signals or playing important roles in DDR signaling by themselves.

### Viral Infections

Viral infections, including the human T-cell leukemia virus type 1 (HTLV), hepatitis C virus (HCV), and cytomegalovirus (CMV) (34–36) are associated with RA development. It has long been known that viral infection pathways represent potent antiviral defense mechanisms that may be disabled upon viral penetration in the host cells. However, viruses also can harness DDR activation by taking control of specific host proteins in the DDR pathway to aid viral replication. Direct evidence regarding how the virus-modified DDR pathway in RA-associated T cells has yet not been obtained; however, T cells derived from RA patients mimic the biological effects of HCV infection in T cells, including cell susceptibility to apoptosis, attenuating the activation of ATM and MRE11A (37). HTLV-1 is a retrovirus associated with RA pathogenesis (38, 39). Upon entering T cells, HTLV-1 expresses Tax and the protein concentration of Tax is several fold higher in the blood of RA patients than in healthy donors (40). Tax is essential for viral replication through deregulation of DDR pathways. The dampened ATM kinase and reduced association of MDC1 with the repair foci have also been reported in Tax-positive cells, which may serve as the mechanism for insufficiency of ATM activity and DNA foci formation in RA-associated T cells (41). Moreover, Tax upregulates c-FLIP and inhibits the apoptosis caused by the CD95 death receptor, a phenomenon also observed in RA-associated T cells (42, 43). Recently, a study reported that the mitochondrial DNA damage activates cytosolic antiviral signaling by promoting interferon production after a herpes virus infection (44, 45). In RA patients, T cells chronically infected with CMV also express large amounts of IFN-γ (46, 47), suggesting that mitochondrial DNA damage signaling may exert synergistic effects with canonical DNA damage stress signaling to trigger antiviral immune response in RA-associated T cells.

### Environmental Events

Constant environmental assaults are inflicted on human T cells inducing DNA damage. Several environmental factors affect either RA susceptibility, or the pathophysiology of RA, and several such environmental factors have been identified. Increased caffeine intake, a history of heavy smoking, and exposure to air pollution are all associated with increased rheumatoid factors. Caffeine reportedly uncouples the cell-cycle progression from DNA repair via the mechanism of directly repressing the G2/M checkpoint by inhibiting ATM kinase (48, 49). However, reports regarding the effects of caffeine are controversial. A cohort study comparing the effects of caffeinated, decaffeinated, and total coffee intake in women did not support the distinct association between caffeinated coffee or caffeine intake with RA pathogenesis (50).

Complex environmental carcinogens, such as tobacco smoke also serve as genotoxic agents that trigger H2AX phosphorylation and DDR signals (51). A mechanistic study reported that citrullinated antigens, the self-antigen for ACPA autoantibody of RA patients, are formed in individuals with a history of smoking via activation of peptidylarginine deiminase at the sites of inflammation (52). A subsequent study reported that nicotine in tobacco is a strong inducer of *p53* gene mutations (52). Nicotine also promotes neutrophil extracellular traps (NETosis) production in the neutrophil of RA patients, which play an important role in RA-associated inflammation. The formation of NETosis need a systematic process for DNA which regulated by DDR signals (53, 54). Those studies suggest that DDR benefit the nicotine-regulated NETosis development and the DDR signal way in T cell and neutrophil of RA patients may be different.

## Impaired DDR Signals Induce the Disease-Related Phenotypes in RA-Associated T Cells

Naïve T cells adapt to stress conditions via homeostatic replication to compensate for the loss of T cells, concurrent with a reduction in the diversity of the T cell repertoire (16–19). In RA disease, T cells adapt to the stress accompanied by modification of the DDR pathway, characterized by telomere shortening, accumulation of DNA damage and the expansion of CD4^+^CD28^−^ end-differentiated T cells. These factors contribute to chronic, histolytic T cell genesis, which is critical for RA progression. This section discusses mechanisms underlying the induction of disease-related changes in T cell function in RA, owing to deficits in the DDR signal pathway.

### The DDR Pathway and T Cell Aging

**Figure 1:**
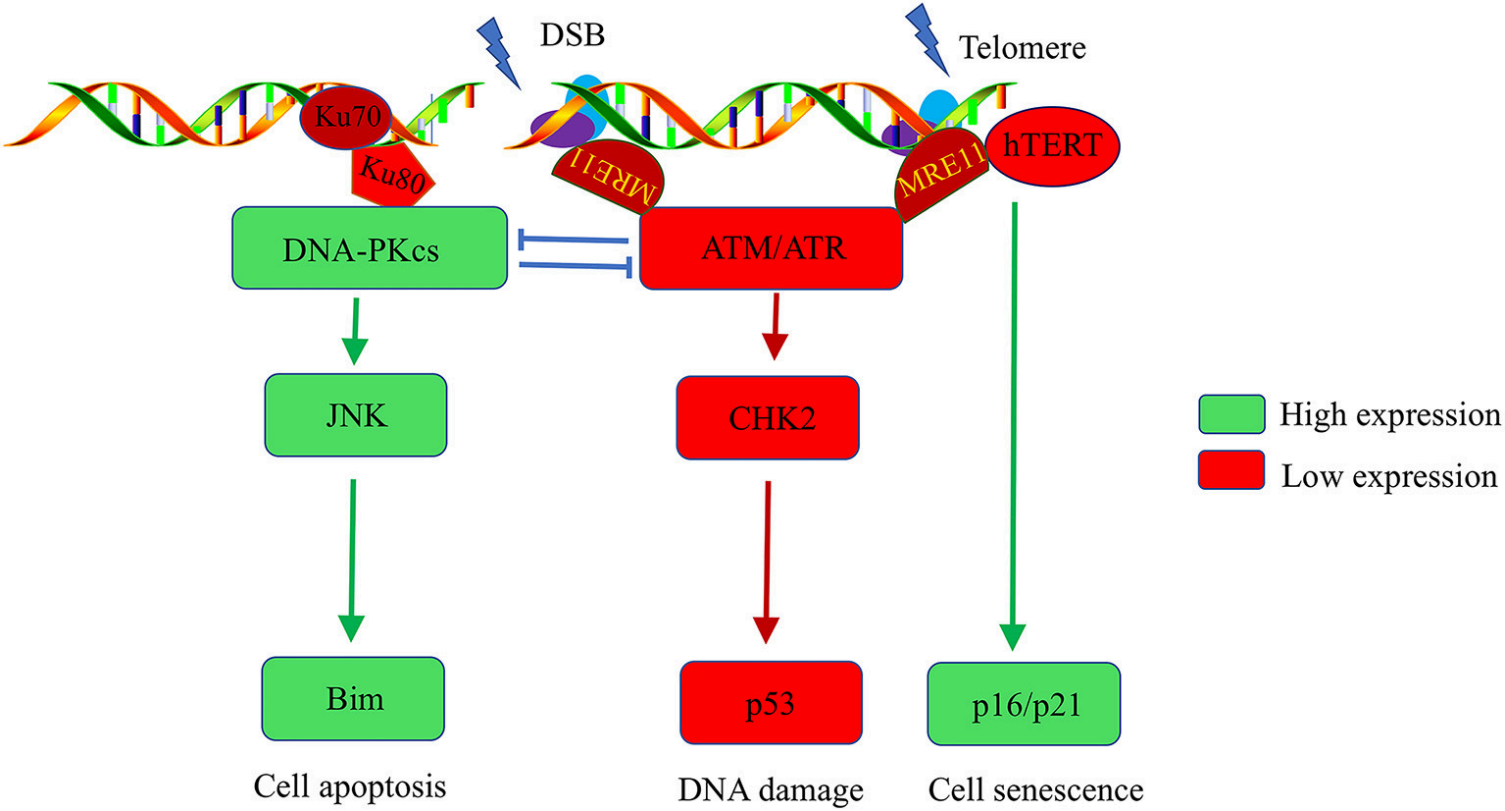
Abnormalities in the DNA damage repair pathway in T cells of rheumatoid arthritis patients. DNA stability is critical for the function of T cells. Naïve T cells from RA patients have ATM deficiency, which impairs the cells' ability to detect and repair DNA lesions. T cells alternatively upregulate DNA-PKcs. The activated DNA-PKcs-JNK-Bim axis eventually forces naïve T cells into apoptosis. Moreover, there is insufficient activation of the ATM kinase, hTERT, and MRE11A in RA-associated T cells after T cell receptor stimulation, which accelerates the G2/M checkpoint bypass and hyperproliferation. Unrepaired DNA breaks and telomere erosion finally induce T cell death and senescence. To compensate for the T cell pool, naive cells are forced into homeostatic clonal expansion, which facilitates the section of autoreactive T cell.

The T cells of RA patients exhibit premature aging and sensitivity to apoptosis, which are characteristics of lymphocytes in individuals with genetic progeroid syndromes. RA patients also recapitulate the features of genetic progeroid syndromes with defects in chromatin maintenance and cardiovascular diseases. The primary contributors to the pathogenesis of genetic progeroid syndromes are hypomorphic mutations in key DNA repair molecule, suggesting that T cells age in RA may be owing to impaired DNA repair. Further studies have confirmed that T cells from RA patients have the ATM-deficient naïve T cells, which fail to sense and repair DNA lesions induced by genotoxic stress (11). Comet assays revealed elongated T cell tails in RA patients compared with healthy controls. Reconstitution of ATM rescues the ability of DNA repair and clears DNA damage in RA-associated T cells (55). Mechanism Studies on DDR signals in RA patients reported that ATM transcripts regulate MRE11A, NBS1, RAD50, and p53 expression. ATM deficiency-mediated impairment of MRN complex in RA T cells fails to detect damaged DNA and subsequently attenuate the recruitment of ATM to damaged DNA foci, thereby leaving DBS unrepaired (11–13). Persistent DBS alternatively upregulates DNA-PKcs, in the NHEJ repair pathway. However, the first step in the NHEJ pathway is the detection of fragmented DNA by Ku70/Ku80, following recruit DNA-PKcs to DNA repair foci to form functional DNA-PK. In T cells of RA patients, DNA-PKcs are upregulated, accompanied with insufficient expression of ku70/ku80. Consequently, instead of repairing damaged DNA by forming DNA-PK, DNA-PKcs participate in the JNK-Bim pathway and induce spontaneous apoptosis in naïve T cells of RA patients (Figure 1) (12).

Moreover, ROS production is inadequate in T cells of RA patients after T cell receptor (TCR) stimulation, thereby blocking the ATM monomer and deterring its dimerization, which is a critical step for ATM kinase activation. ATM activity deficiency accelerates the G2/M checkpoint bypass and promotes apoptosis in RA-associated T cells after activation. Excessive loss of T cells in RA patients make a replicative stress to naive cells. To maintain the size of T cell pool, naive cells are forced to undergo homeostatic clonal expansion, thereby accelerating aging of the T cell repertoire and promoting oligoclonal expansion. Consequently, cell repertoire diversity is contracted which favor the autoreactive T cell selection and RA development (12).

### The DDR Pathway and Telomere Intactness of T Cells

Telomeres are the most fragile sites to DNA damage stress in chromosomes. Telomere damage has several-fold more severe effects on the cell than does damage at other chromosomal sites (56). Telomere stability and the intactness of telomeres are critical for maintaining cell longevity and proliferation. Naive T cells from RA patients have shortened telomeres (21). Several potential mechanisms underlie non-age-dependent acceleration of telomeric erosion found in RA T cells. First, telomeric erosion may reflect an increased proliferative frequency of naive T cells in RA. Second, telomeric lengthening and maintenance are facilitated by telomerase, which is composed of a catalytic protein unit known as human telomerase reverse transcriptase (hTERT), and an RNA template complementary to the telomeric DNA (hTR) (57). T cells from RA patients have reduced hTERT expression and fail to upregulate telomerase activity after TCR stimulation. Insufficient telomerase also accelerates telomere shortening in T cells of RA patients (14). Moreover, telomeric shortening could be a consequence of deficits in DNA repair. In the G2 cell cycle phase, telomere overhang is recognized by ATM-regulate DDR signals that promote telomere shelterin-complex reconstitution, which is critical for telomere protection. ATM defects leave telomeres unprotected, subsequently trigger chromosome fusions and instability (58–60). Li et al. recently reported that reduced activity of MRE11A, a component in MRN complex, induces a cellular senescence module by promoting telomere uncapping and upregulation of cell senescence markers, such as p16, p21, and the cell surface receptor CD57 in RA-associated T cells (Figure 1). Moreover, MRE11A deficiency is directly implicated in the proinflammatory properties and tissue invasive in RA-associated T cells (13).

### The DDR Pathway and T Cell Metabolism

Regulation of nutrient uptake and utilization in T cells is critical for signaling, differentiation, function, and the fate of T cells (61, 62). Metabolically, naive T cells from RA patients cannot supply enough energy to adapt to the demands of TCR stimulation. Insufficient induction of 6-phosphofructo-2-kinase/fructose-2,6-bisphosphatase 3 (PFKFB3), a rate-limiting glycolytic enzyme, leads to a deficient glycolysis flux in RA-associated T cells. Interestingly, PFKFB3 is also involved in DNA HR repair. Upon detecting the DSBs, PFKFB3 rapidly recruited to DNA repair foci via the MRN-ATM-γH2AX-MDC1 complex and participates in the DNA repair process (63), which indicating a strong connection between DDR signals and PFKFB3 regulated cellular metabolism.

In RA-associated T cells, reduced PFKFB3 decreased lactate/ATP levels and increased NADPH/biosynthetic precursor levels compared to T cells from healthy individuals (10, 64–66). The equivalent^low^NADPH^high^ condition in RA patients fails to appropriately balance mitochondrial ROS production and the cellular anti-oxidant machinery. Thus, ROS production is reduced in RA-associated T cells (10). Insufficient ROS prevents ATM activation and disrupts the G2/M checkpoint after T cells stimulation. Moreover, ATM is critical for mitochondrial biogenesis, ATM deficiency may be associated with aberrant mitochondria in T cells of RA patients (67, 68).

Concerning lipid metabolism, RA-associated T cells have increased fatty acid biosynthesis and decreased fatty acid oxidation. Usually, AMPK-mediated p53 activation negatively regulates lipid synthesis by suppressing mTOR (69). AMPK-p53 function is impaired in T cells of RA patients, which potentially explaining the enhanced mTOR activity, decreased fatty acid oxidation, and increased fatty acid synthesis observed in RA T cells (64, 70). How is metabolic dysfunction in those cells linked to RA pathogenesis? A recent study reported that an ATP^low^pyruvate^low^ environment in RA patients induces adequate expression of TKS5, a podosome scaffolding protein, which triggers fatty acid biosynthesis and promotes T cell locomotion capabilities. These factors, together, promote the formation of tissue-invasive membrane structures in RA-associated T cells and facilitates the infiltration of inflammatory T cells in RA patients into non-lymphoid tissue sites (71).

Many key regulatory molecules in DDR pathways associated with RA development also play a role in cellular metabolism (72–74). Sirtuins are a class of NAD^+^-dependent deacetylases, which are critical for maintaining genomic homeostasis. Sirtuins control energy metabolism by regulating PGC-1α and forkhead transcription factors (FOXO) to supply the metabolites required for DNA repair (75, 76). The DNA damage repair enzymes, PARPs and SENPs, play important roles in metabolic regulation owing to their influence on mitochondrial function and oxidative metabolism (77, 78). Future studies are expected to elucidate the intrinsic associations between DDR signals and metabolic homeostasis in the T cells of RA patients.

### The DDR Pathway and T Cell Differentiation

CD4^+^ T cells differentiate into specific subtypes with unique immune functions and secretion of specific cytokines; classical T cell subsets include Th1, Th2, Th17, and Treg (79). DDR signaling abnormalities in RA may accelerate synovial inflammation by affecting T cell differentiation. Hyperproliferative T cells in RA have biased differentiation patterns toward the Th1 and Th17 fate (10, 13). Therefore, Th1-associated cytokines IFN-γ and Th17-associated cytokines IL-17 are upregulated in the serum and synovial fluid of RA patients and serve as the primary inducer of the inflammatory response (80, 81). Both ATM and p53 reportedly regulate differentiation of T cells in RA (10, 82). The insufficient ATM-p53 function in T cells from RA patients shifts the differentiation of naïve CD4^+^ T cells toward the Th1 and Th17 effector lineages rather than Treg cells, thereby generating an arthrogenic effect of T cells and imposing a hyperinflammatory phenotype in the synovial tissue (10, 82). Moreover, ATM (-/-) mice have abnormal CD8, T cell immune responses with defective expansion, contraction, and differentiation bias to the effector-to-memory pathway. Mechanistic studies reported that CD8^+^ T cells with ATM deletion have hyperactive mTORC1 signaling, which results in inflammatory cytokine production and memory T cell development and acceleration of the autoimmune genesis in RA patients (83). Recently, a new subset of T peripheral helper (Tph) cell populations in RA patients with PD-1^hi^CXCR5^−^ Bcl6^low^ was identified, which infiltrating inflamed synovia by expressing the chemokine receptor, CCR2, and augmenting synovial B cell responses (84). Interestingly, p53 could repress the CXCR5 chemokine receptor gene via attenuation of NF-κB activity (85), and reduction of BCL6 enhance the DDR pathway. Those studies indicate that DDR pathway exert a strong influence in Tph formation and further studies are expected to examine the DDR signals in Tph subset of RA patients.

## Perspective

**Figure 2:**
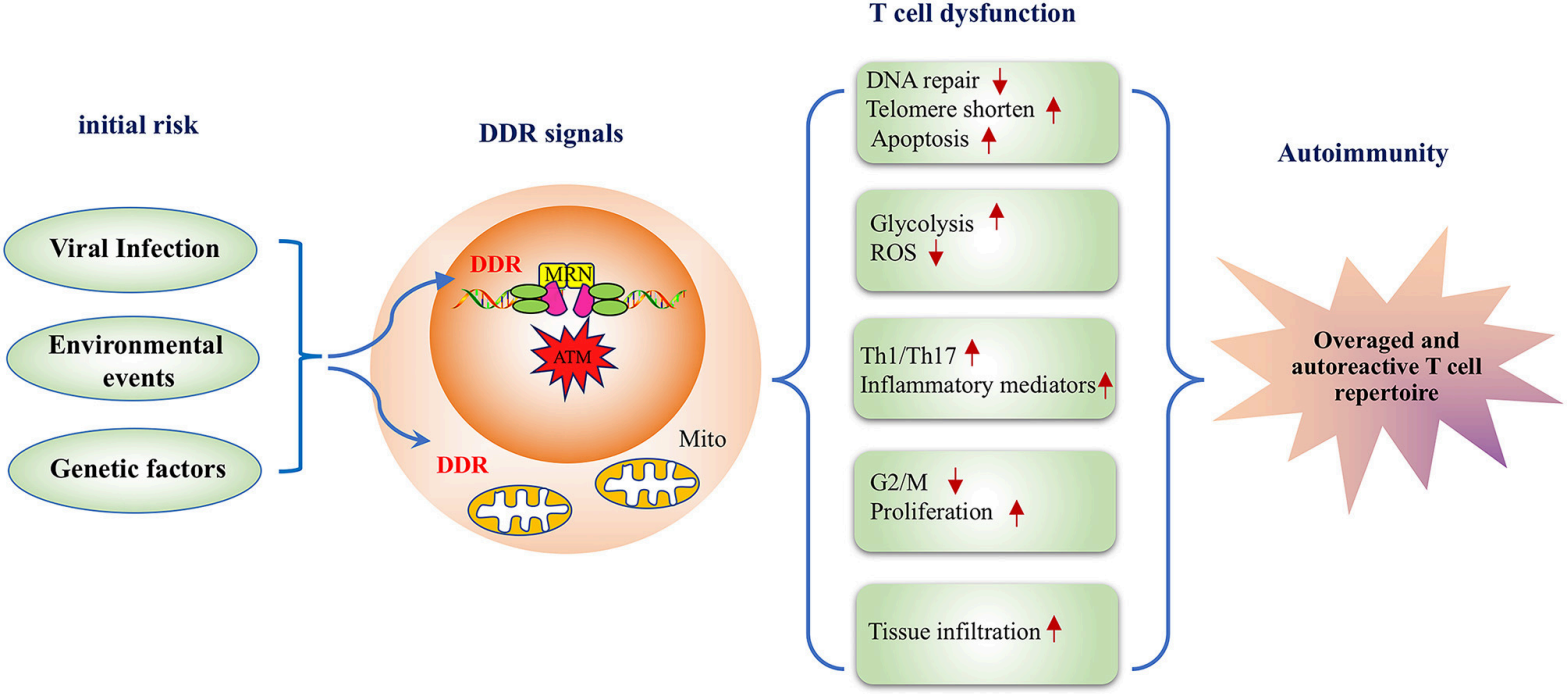
A model depicting the DNA damage response pathway inducing disease-related phenotypes in T cells in rheumatoid arthritis. RA risk factors, including viral infection, environmental events, and genetic factors lead to defective DDR signals in T cells, and the inadequate DDR responsiveness subsequently alters T cell functionality in multiple aspects. First, deficiency in DNA repair influences T cell survival resulting in a lower apoptotic threshold. Second, DDR is implicated in the metabolic regulation of RA-associated T cells. Moreover, an impaired DDR response biases T cell differentiation to inflammatory effector T cells. Therefore, DDR deficiency in RA-associated T cells represents a key mechanism for the pathogenesis of RA with transitional genetic and environmental factors to RA pathogenesis-related T cell dysfunction.

Significant advancements have facilitated the understanding of the DDR pathway and RA; however, much remains to be learned. This review discussed studies on the molecular mechanisms underlying the effects of environmental, viral, and genetic risk factors of RA on DDR signaling and summarized how the key players in DDR signaling induce RA-related phenotypes such as apoptosis, aging, metabolic disorders, and an imbalance in the differentiation of T cells. Based on these studies, the present review suggests that DDR signaling plays an important role in the autoimmune pathogenesis of RA (Figure 2). To further clarify the role of DDR signaling in the development of RA-related autoimmunity, prospective studies on the preclinical and early phases of RA development are warranted. Those studies will provide a novel avenue to further the understanding of RA.

## Author Contributions

The author confirms being the sole contributor of this work and has approved it for publication.

### Conflict of Interest Statement

The author declares that the research was conducted in the absence of any commercial or financial relationships that could be construed as a potential conflict of interest.

## Abbreviations

DDR: DNA damage response
RA: Rheumatoid arthritis
MRN: MRE11A-Rad50-NBS1
ATM: ataxia telangiectasia mutated
PIKK: phosphoinositide-3 kinase-like kinase
DSBs: DNA double strand breaks
ssDNA: single-stranded DNA
HR: Homologous recombination pathway
NHEJ: non-homologous end joining
GWASs: genome-wide association studies
SNPs: single nucleotide polymorphisms
ACPA: anticitrullinated protein antibodies
HTLV-1: Human T-cell leukemia virus type 1
HCV: hepatitis C virus
CMV: Cytomegalovirus
NETs: neutrophil extracellular traps
hTERT: telomerase reverse transcriptase
hTR: RNA template complementary to the telomeric DNA
TCR: T cell receptor
PFKFB3: 6-phosphofructo-2-kinase/fructose-2,6-bisphosphatase 3
PGC: peroxisome proliferator-activated receptor-γ coactivator
PPP: pentose phosphate pathway
FOXO: forkhead transcription factors
Tregs: regulatory T cells.
